# Cholinergic regulation of neuroinflammation: linking microglia, immunometabolism, and neuromodulation

**DOI:** 10.3389/fimmu.2026.1837643

**Published:** 2026-06-19

**Authors:** Hui Guo, Ziyu Yang, Long Cheng

**Affiliations:** Department of Neurology, Xianyang Hospital of Yan’an University, Xianyang, China

**Keywords:** cholinergic signaling, immunometabolism, microglia, neuroinflammation, vagus nerve stimulation

## Abstract

Neuroinflammation is increasingly recognized as a core pathological process in various neurological diseases, including neurodegenerative disorders, stroke, autoimmune demyelinating diseases, and acute brain dysfunction associated with systemic inflammation. Among its regulatory mechanisms, the cholinergic anti-inflammatory pathway links neural activity with immune regulation. However, its neurological relevance extends beyond the classical peripheral vagus nerve-mediated inflammatory reflex. Within the central nervous system, cholinergic signaling interacts with resident immune cells, particularly microglia, and influences inflammatory tone, neuronal vulnerability, and tissue repair. Recent advances in immunometabolism further suggest that metabolic reprogramming may bridge cholinergic signaling and microglial inflammatory phenotypes. In this review, we discuss the role of cholinergic regulation of neuroinflammation from three interrelated perspectives: microglia as the hub of core cells, immune metabolism as the basis of mechanism, and neural regulation as the frontier of transformation. We first reviewed the cholinergic system and its role in neuroimmune communication, then discussed how cholinergic signals shape microglial state and metabolic process, and finally evaluated its disease-specific evidence in Alzheimer’s disease, Parkinson’s disease, stroke, multiple sclerosis and acute inflammatory brain dysfunction. We will also discuss pharmacological and bioelectronic methods, including targeting cholinergic receptors and vagus nerve stimulation, as emerging therapeutic strategies. By integrating cholinergic biology, microglial heterogeneity, and metabolic reprogramming, this review proposes an updated framework for understanding neuroinflammation in neurology, and highlights the future opportunities for precise neuroimmune intervention.

## 1 Introduction

Neuroinflammation is increasingly recognized as the common core pathological process of a variety of nervous system diseases, including neurodegenerative diseases, stroke, autoimmune demyelinating diseases, traumatic brain injury, and acute brain dysfunction associated with systemic inflammation (1, 2). Although a controlled inflammatory response is essential for host defense, tissue repair, and central nervous system (CNS) homeostasis monitoring, persistent or dysfunctional neuroinflammation can lead to synaptic dysfunction, neuronal damage, and progressive neurological decline (3). Therefore, neuroinflammation is no longer regarded as a secondary result of CNS injury, but an important active determinant of disease progression and treatment response (4).

Microglia, as the resident immune sentinel of CNS, play a particularly important role in the main cellular mediators of neuroinflammation (4). In the face of protein aggregates, ischemia, systemic inflammatory signals and age-related tissue stress, microglia will undergo dynamic phenotypic and functional changes, which may not only promote repair, but also aggravate tissue damage (5, 6). At the same time, it is currently believed that microglia activation is highly heterogeneous, and its state is shaped by disease background, local microenvironment and metabolic state, rather than a simple binary classification (6). This complexity makes microglia become the core research object to understand how neural signals affect the outcome of inflammation in the brain.

One of the most influential conceptual developments in this field is the cholinergic anti-inflammatory pathway (CAP), initially described as an inflammatory reflex mediated by the vagus nerve, which can inhibit the production of cytokines through acetylcholine dependent signals (7). In its classic expression, cap emphasizes the interaction between vagus nerve activity, acetylcholine release and α 7 nicotinic acetylcholine receptor (α 7nachr) - dependent peripheral immune cell regulation (8). However, the correlation between cholinergic signal and neuroinflammation is far beyond this peripheral reflex model. While the α7 nicotinic acetylcholine receptor has received the greatest attention, other nicotinic and muscarinic receptor subtypes may also contribute, as will be discussed later. In CNS, cholinergic pathway can affect glial cell activation, neuron glial cell communication, neurovascular function and inflammatory state, suggesting that cholinergic immune regulation should be considered as part of a broader neuroimmune network (8, 9).

More and more evidences show that microglia may be an important cellular hub for cholinergic signaling to regulate neuroinflammatory response (9). Astrocytes, which also express cholinergic receptors and participate in metabolic support and neuroimmune crosstalk, are increasingly recognized as additional contributors, as detailed in later sections. Microglia expressed cholinergic receptors, especially α 7nachr; Cholinergic stimulation is associated with changes in cytokine release, inflammasome activity, oxidative stress and phagocytosis (8, 9). However, it is not enough to fully explain the diverse effects of cholinergic regulation observed in different nervous system diseases only from the signal transduction at the receptor level. Therefore, immune metabolism has gradually attracted attention, which provides a mechanism framework for understanding how metabolic programs regulate the phenotype and function of immune cells (10, 11). In microglia, inflammatory activation is closely related to metabolic remodeling such as glycolysis, mitochondrial function, lipid metabolism and redox balance, and these pathways may be directly or indirectly regulated by cholinergic signaling (11). Recent conceptual advances have highlighted central metabolic sensors—such as AMPK, mTOR, and HIF-1α—as key integrators of immune and metabolic signals in neurodegenerative conditions including Alzheimer’s disease, Parkinson’s disease, and amyotrophic lateral sclerosis.

This emerging intersection among cholinergic signaling, microglial biology, and immunometabolism is particularly relevant in neurology. In disorders such as Alzheimer’s disease, Parkinson’s disease, stroke, and multiple sclerosis, and amyotrophic lateral sclerosis, cholinergic dysfunction coexists with persistent neuroinflammation and altered microglial states (2, 12). At the same time, nerve regulation strategies such as vagus nerve stimulation and drug intervention targeting cholinergic pathway are attracting more and more attention as potential anti-inflammatory treatment methods (7, 12). These advances suggest that reexamining the cholinergic anti-inflammatory framework from a CNS centric perspective may provide new insights into disease mechanisms and treatment opportunities.

In this review, we will discuss the regulation of cholinergic on neuroinflammation from three interrelated perspectives: microglia as the hub of core cells, immune metabolism as a mechanism bridge, and neural regulation as the frontier of transformation. We first outline the cholinergic system and its role in neuroimmune communication, then explore how cholinergic signaling reshapes the inflammatory phenotype and metabolic processes of microglia, and finally evaluate the relevance of these mechanisms in major neurological diseases and emerging treatment strategies.

## 2 The cholinergic system and neuroimmune communication

**Figure 1:**
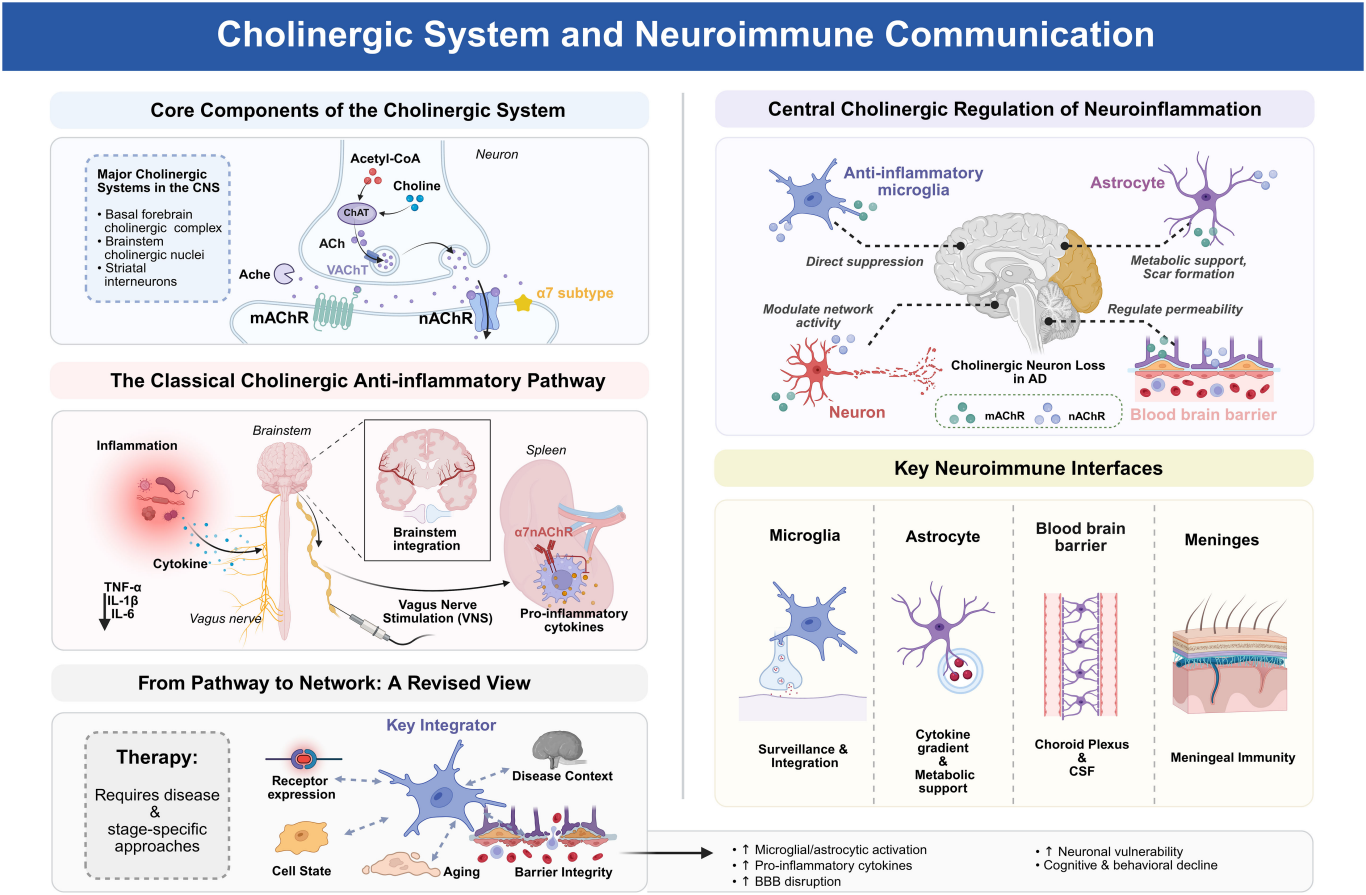
Overview of the cholinergic system and its role in neuroimmune communication during neuroinflammation.

The cholinergic system is traditionally understood as a neurotransmitter network with acetylcholine (ACh) as the core, which plays an important role in cognition, arousal, attention, autonomic nerve regulation and neuromuscular transmission (13, 14). However, in recent years, its function has gone beyond the classical neurotransmission category and extended to a wide range of immunoregulatory effects in peripheral tissues and CNS (7, 14). This broader perspective is particularly important for neuritis, because cholinergic signals may affect resident immune cells, barrier structures, autonomic efferent and systemic inflammatory feedback loops (8, 15) (**Figure 1**).

### 2.1 Core components of the cholinergic system

ACh is synthesized from choline and acetyl-CoA by choline acetyltransferase (ChAT), stored in vesicles by vesicular acetylcholine transporter (VAChT), released into synaptic or extrasynaptic spaces, and rapidly degraded by acetylcholinesterase (AChE) or butyrylcholinesterase (13, 16). In the CNS, cholinergic neurons are concentrated in several anatomically and functionally distinct systems, including the basal forebrain cholinergic complex, brainstem cholinergic nuclei, and striatal interneurons (13, 16). These pathways regulate cortical activation, hippocampal function, sleep-wake transitions, motor control, and autonomic integration, all of which are closely linked to inflammatory vulnerability in neurological disease (16, 17).

Cholinergic signaling is mediated through two major receptor classes: nicotinic acetylcholine receptors (nAChRs), which are ligand-gated ion channels, and muscarinic acetylcholine receptors (mAChRs), which are G-protein-coupled receptors (17, 18). Among nAChRs, the α7 nicotinic acetylcholine receptor (α7nAChR) has received the greatest attention in immune regulation because of its anti-inflammatory effects in macrophages, microglia, astrocytes, and other cell types (7, 8). Muscarinic receptors have been less extensively studied in neuroimmune regulation, but emerging evidence suggests that they may also influence microglial function, astrocytic signaling, and neurovascular responses in context-dependent ways (18). Thus, the immunological relevance of the cholinergic system likely extends beyond α7nAChR alone. mAChRs and non−α7 nAChR subtypes are increasingly recognized as additional contributors to cholinergic neuro−immune communication. In preclinical models, activation of mAChRs attenuates microglial pro−inflammatory phenotypes and reduces cytokine/chemokine expression in the CNS (19). Moreover, a positive allosteric modulator of the M1 mAChR subtype reduces microglial and astrocytic activation in an Alzheimer’s disease mouse model (20). Among non−α7 nAChRs, the α4β2 subtype has been shown to suppress neuroinflammation via the JAK2−STAT3 signaling pathway in ischemic models (21), while α9-containing nAChRs differentially modulate autoimmune neuroinflammation in experimental autoimmune encephalomyelitis (22). The downstream signaling pathways of these receptors (e.g., JAK2-STAT3 for α4β2 nAChR) will be discussed in the context of immunometabolism (Section 4), while their therapeutic potential is addressed in the translational section.

### 2.2 The classical cholinergic anti-inflammatory pathway

The cholinergic anti-inflammatory pathway was originally described as a neural reflex that links the nervous system to peripheral immune regulation (7). In this model, inflammatory signals are sensed by afferent vagal pathways and integrated within the brainstem, followed by efferent autonomic output that ultimately suppresses cytokine production through cholinergic signaling (7). Although the precise anatomical structure of this loop is still an active research field, its most clear mechanism of effect involves the activation of ACh mediated innate immune cells, especially α 7nachr on macrophages, thereby inhibiting the release of pro-inflammatory cytokines (8, 23).

This framework provides a conceptual breakthrough, proving that inflammation can be regulated through a clear neural circuit rather than relying solely on humoral immune pathways (7, 23). It also opened the door to therapeutic neuromodulation strategies, such as vagus nerve stimulation (VNS), aimed at controlling inflammatory disease (24, 25). However, the classical cap model is mainly based on the research of peripheral immunology and Autonomic Neuroscience, so it cannot fully cover the complexity of cholinergic regulation in nervous system diseases; In these diseases, the inflammatory process is jointly shaped by the unique resident cell population, tissue structure and blood-brain barrier of CNS (8, 25).

### 2.3 Central cholinergic regulation of neuroinflammation

In CNS, the cholinergic regulation of inflammation cannot be simply regarded as an extension of the peripheral cap model. The central cholinergic circuit affects the inflammatory state through a variety of mechanisms, including direct receptor-mediated action on glial cells, regulation of neuronal excitability, regulation of neurovascular coupling, and control of autonomic nerve balance (8, 26). Degeneration of central cholinergic neurons, especially basal forebrain cholinergic neurons, is a hallmark of Alzheimer’s disease, which is not only related to cognitive decline, but also related to the enhancement of neuroinflammatory signals and the impairment of tissue toughness (26, 27). SIn Parkinson’s disease, delirium and post-stroke brain injury, studies have also suggested that there is a similar link between cholinergic dysfunction and inflammation (27, 28).

In the brain, cholinergic signals can shape the inflammatory microenvironment at multiple levels. First, microglia and astrocytes express cholinergic receptors and may directly respond to ach or cholinergic agonists (9, 29),; for astrocytes, cholinergic signaling has been shown to influence inflammatory gene expression, metabolic support to neurons, and glial scar formation, with the net outcome depending on the specific receptor subtype and pathological context (30). Secondly, neurons themselves can also regulate immune state by changing network activity, transmitter release and local steady-state signals (31). Third, cholinergic signals affect endothelial cells, perivascular macrophages and barrier related structures, thereby affecting leukocyte entry, vascular permeability and CNS exposure to peripheral inflammatory mediators (31, 32). In parallel, through its modulation of autonomic balance, cholinergic signaling indirectly shapes the metabolic and inflammatory state of both microglia and astrocytes, an aspect that will be further addressed in the context of immunometabolism. These multi-level effects suggest that the regulation of cholinergic on neuroinflammation is more suitable to be understood as a distributed neuroimmune system than a single pathway.

### 2.4 Neuroimmune interfaces relevant to cholinergic signaling

There are several key neuroimmune interfaces in the brain, and cholinergic signals may play an anti-inflammatory or immunomodulatory role through these interfaces (32). The most obvious is the microglial network, which continuously monitors the brain parenchyma and rapidly integrates neural and inflammatory inputs (4, 9). Astrocytes can respond to neurotransmitters and immune mediators at the same time, and may act as amplifiers or regulators of cholinergic effect by shaping cytokine gradients, metabolic support and synaptic microenvironment (33). The blood-brain barrier and neurovascular units represent another key interface, because endothelial cells and perivascular immune cells can convert neural activity into vascular inflammatory response (31, 33).

In addition, meningeal immunity, cerebrospinal fluid oriented ventricles and choroid plexus have been considered as important immune communication areas, which can connect the peripheral inflammatory state with the immune dynamics of the central nervous system (34). These compartments are particularly important in aging, infection, and neurodegeneration, because in these cases, peripheral inflammatory signals may be easier to enter the CNS regulatory system. Cholinergic signals may directly or indirectly affect these interfaces, although the mechanism is not completely clear, and it is likely to vary according to the tissue niche and disease status (34, 35). This background dependence is also one of the reasons why the same cholinergic intervention may produce different inflammatory outcomes in different diseases.

### 2.5 From pathway to network: a revised view of cholinergic neuroimmune regulation

Overall, the existing evidence shows that the cholinergic anti-inflammatory pathway in neurology should be redefined as a broader cholinergic neuroimmune regulatory network (8, 25). The traditional vagal center model still has important basic value, but it is not enough to explain all the cholinergic effects observed in CNS diseases. In nervous system diseases, cholinergic regulation is likely to result from the interaction between central cholinergic tension, peripheral autonomic nerve signals, glial cell receptor biology, barrier function and disease-specific immune background (26, 36).

This broader perspective is of great significance for both mechanism and treatment. At the mechanism level, it suggests that receptor expression alone is not enough; Cell status, tissue niche, metabolic configuration and loop background may determine whether cholinergic input is inhibited, redirected, or unable to change inflammation (10, 36). At the treatment level, it means that successful neural regulation is likely to require disease-specific and stage specific methods, rather than a general “activate CAP” strategy (24, 36). These considerations naturally lead to the next important question: which CNS cells are the most important integrators of cholinergic signals in the process of neuroinflammation? More and more evidences point to microglia. Importantly, the expression of cholinergic receptors is not static but dynamically regulated by aging, disease stage, and the functional state of glial cells, implying that the outcome of cholinergic interventions may depend critically on cellular and tissue context (37).

## 3 Microglia as the central cellular hub of cholinergic neuroinflammation control

**Figure 2:**
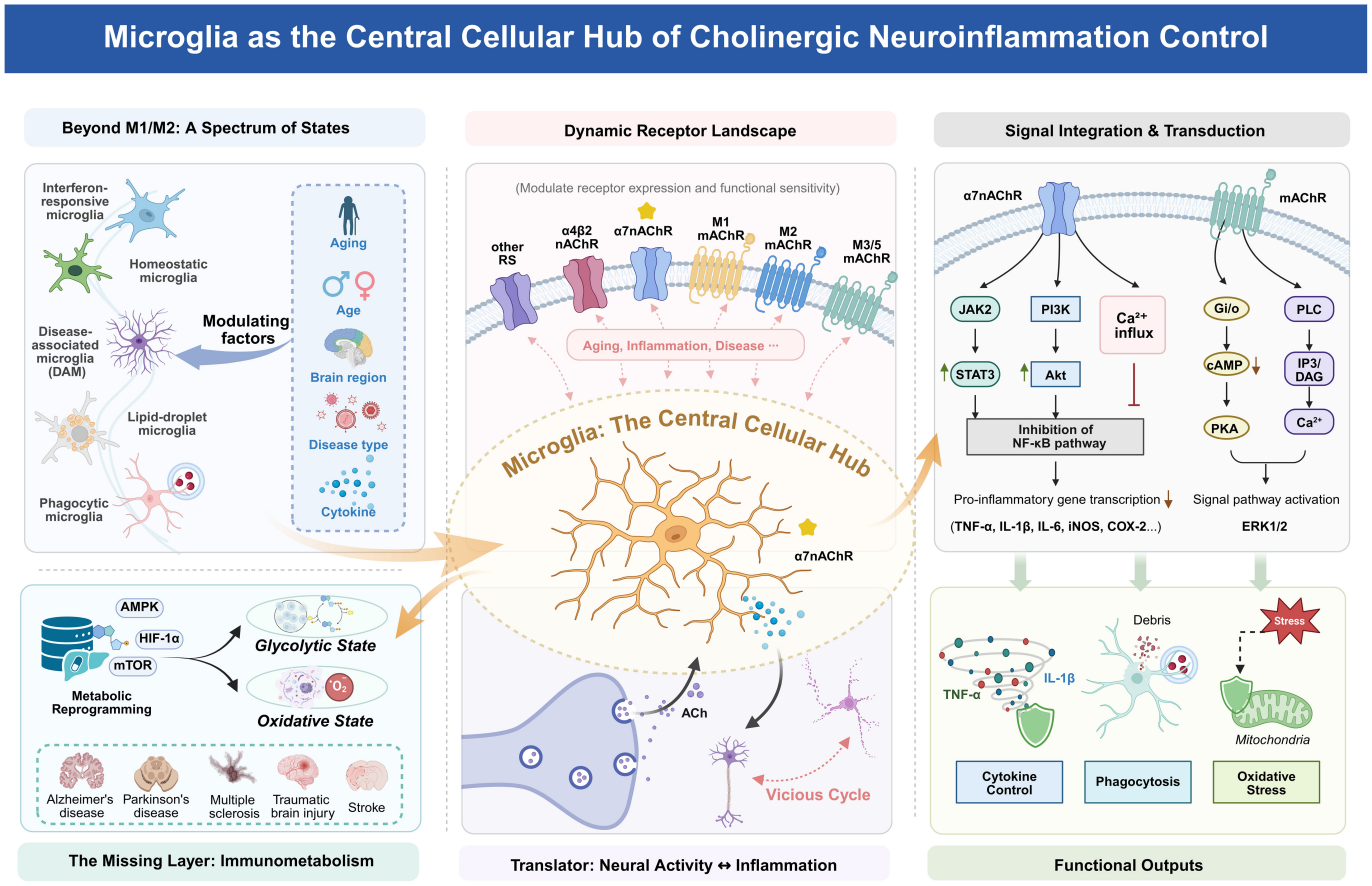
Microglia as a central hub of cholinergic control in neuroinflammation.

Microglia are increasingly recognized as the principal cellular interface through which cholinergic signaling may regulate neuroinflammatory responses in the CNS (38) (**Figure 2**). As resident myeloid cells of the brain, microglia are strategically positioned to sense neuronal activity, tissue damage, metabolic stress, vascular dysfunction, and systemic inflammatory signals (4, 39). Their functions extend well beyond classical immune surveillance and include synaptic remodeling, phagocytic clearance, trophic support, regulation of myelin homeostasis, and coordination of glia-neuron communication (12, 39). Because these functions can shift rapidly across physiological and pathological conditions, microglia have become central to current models of how neural circuits influence inflammatory outcomes in the brain (10, 40).

### 3.1 Microglial heterogeneity beyond the M1/M2 paradigm

Early studies frequently described microglial activation using the binary framework of pro-inflammatory “M1-like” and anti-inflammatory “M2-like” states. Although this model helped establish the concept of microglial polarization, it is now widely considered insufficient to capture the complexity of microglial biology *in vivo (*10, 11). Single-cell and spatial approaches have shown that microglia occupy a spectrum of transcriptional, functional, and metabolic states shaped by age, brain region, sex, disease type, and disease stage (11, 31). Homeostatic microglia, disease-associated microglia, interferon-responsive microglia, lipid-droplet-accumulating microglia, phagocytic microglia, and injury-associated states have all been described across neurological conditions (12, 31).

This heterogeneity is particularly important when considering cholinergic regulation. Cholinergic signaling is unlikely to produce consistent anti-inflammatory effects in all microglial states; On the contrary, its impact may depend on receptor availability, intracellular signal bias, local metabolic conditions and the surrounding cytokine environment (41). Therefore, microglia should not be regarded as passive receptors of cholinergic tension, but as integrators with background sensitivity that can transform neurotransmitter inputs into different inflammatory outputs. This view helps explain why cholinergic interventions can appear highly effective under certain experimental conditions, while producing different outcomes across disease models and clinical contexts (36, 42). Also, aging and sex are increasingly recognized as major determinants of microglial heterogeneity and responsiveness to cholinergic signals. Aged microglia exhibit distinct transcriptional and metabolic profiles, like heightened inflammatory priming and reduced mitochondrial resilience, that may alter their sensitivity to α7nAChR-mediated regulation (43). Similarly, sex differences in microglial gene expression and cholinergic receptor levels have been reported in several neurological disorders, suggesting that therapeutic outcomes of cholinergic interventions may vary between males and females (44).

### 3.2 Cholinergic receptor expression on microglia

Microglia express a variety of components of the cholinergic signaling system, of which α7nAChR is the receptor subtype most closely related to anti-inflammatory signaling and most well studied (8, 9). In a variety of experimental systems, the activation of α7nAChR on microglia is related to inhibiting the release of pro-inflammatory cytokines, reducing the activation of inflammatory bodies and reducing oxidative stress-related damage (36, 42). Although α7nAChR is the main object of attention in the literature, the evidence also suggests that other nicotinic receptor subtypes and muscarinic receptors may be involved in the regulation of microglial behavior (18, 45). These pathways have not yet been fully elucidated, but may be involved in the state specific or region specific effects of cholinergic regulation.

Importantly, microglial receptor expression is dynamic rather than fixed. Aging, neurodegeneration, ischemia, systemic inflammation and drug exposure may change the abundance or functional reactivity of cholinergic receptors on microglia (45, 46). This has two meanings. First, the response of disease-related microglia to cholinergic stimulation may be different from that of steady-state microglia. Secondly, the receptor expression profile itself may become a biomarker of inflammatory stage or therapeutic response (47).Understanding these dynamic receptor maps is essential for interpreting experimental results and developing precise cholinergic interventions in nervous system diseases. This dynamic regulation may partly explain why cholinergic interventions that show robust anti-inflammatory effects in some experimental models fail to replicate in others or yield inconsistent outcomes in clinical trials: the target microglial population may have already shifted to a state with different receptor availability or signaling competence (48).

### 3.3 Intracellular signaling pathways linking cholinergic input to microglial phenotype

Cholinergic signaling affects microglial function through a series of intracellular pathways, which converge on inflammatory transcription, cell survival, redox homeostasis and immune response (39, 47). Among them, inhibition of NF-κB signal is one of the most frequently reported mechanisms related to α7nachr activation (8). By weakening the transcription program driven by NF-κB, cholinergic stimulation may reduce the expression of TNF-α, IL-1β, IL-6, inducible nitric oxide synthase and other neurotoxic inflammatory mediators (49). At the same time, cholinergic signaling is also associated with reduced activation of NLRP3 inflammasomes, suggesting that it may limit the maturation and release of IL-1β and IL-18 under inflammatory conditions (50).

Other pathways considered to be involved in the regulation of cholinergic microglia include jak2/stat3, pi3k/akt, MAPK cascade and calcium dependent signals (43, 50, 51),,. These pathways may not only affect the production of cytokines, but also affect phagocytosis, cell survival, mitochondrial integrity, and the interaction between microglia and neurons and astrocytes. In some cases, cholinergic stimulation seems to shift microglia from inflammatory amplification to repair or steady-state function; In other cases, it may mainly limit the intensity of damage induced activation rather than actively drive the restorative phenotype (51). The final results are likely to depend on the timing of intervention, receptor subtypes, the intensity of inflammatory stimulation and the existing state of cells.

### 3.4 Functional consequences of cholinergic regulation in microglia

At the functional level, cholinergic regulation may affect many core aspects of microglial behavior. The first is cytokine control. A consistent theme in different models is that cholinergic stimulation, especially through the α7nachr related pathway, can reduce the production of major pro-inflammatory cytokines and chemokines (36, 52). This effect is particularly important in diseases in which sustained release of microglial cytokines can lead to neuronal dysfunction, spread of glial inflammation and destruction of blood-brain barrier.

The second is the control of phagocytosis and tissue remodeling. Microglia are essential for the removal of debris, synaptic pruning, and the removal of damaged cellular components (12, 53). However, under pathological conditions, phagocytosis may become maladaptive, resulting in excessive synaptic loss or incomplete clearance of toxic substances (53). Cholinergic signals may help recalibrate these functions, although the current evidence is inconsistent and is likely to depend on the disease background (54). In some models, cholinergic activation promotes the regression phase of inflammation by supporting a more efficient and less costly clearance process; In other models, it seems that it mainly inhibits over activation, but does not clearly improve clearance (54).

The third is the regulation of oxidative stress and mitochondrial stress. Microglia in the state of neuroinflammation often produce reactive oxygen species and nitric oxide, which can damage neurons and further amplify inflammatory signals (55). Cholinergic pathway is related to the reduction of oxidative load and the maintenance of mitochondrial function in activated microglia, which suggests that its anti-inflammatory effect may be achieved not only by transcriptional inhibition, but also by improving cell stress tolerance (55, 56). This view is particularly important when considering immune metabolism, because mitochondrial function and bioenergy state are closely related to the phenotype of microglia.

### 3.5 Microglia as translators between neural activity and inflammatory outcome

Microglia have a unique position in transforming cholinergic nerve input into a biologically significant inflammatory outcome. Unlike peripheral immune cells, microglia play a role in the neural circuit, can respond to neurotransmitters and directly affect the synaptic environment (31, 56). This means that the regulation of cholinergic on microglia is not a simple anti-inflammatory switch, but a part of a broader system in which neurotransmission, immune perception and metabolic states intersect. Under physiological conditions, this regulation may help to maintain tissue homeostasis and prevent inappropriate inflammatory escalation. However, in the disease state, cholinergic dysfunction may allow or even amplify the maladaptive microglia state, thus promoting neurodegeneration, repair damage or chronic symptoms (26, 57).

This framework also helps to explain why cholinergic defects and neuritis often coexist in nervous system diseases. The two may not be parallel but unrelated abnormalities, but are coupled through microglial dysregulation. Loss of cholinergic tension may remove the important “brake” on inflammatory activation, while chronic microglia activation may further destroy cholinergic signals through synaptic damage, nutritional imbalance and network dysfunction (28, 57). This two-way interaction may form a mutually reinforcing cycle of inflammation and neuronal damage. Furthermore, microglial responses to cholinergic signals are modulated by close crosstalk with astrocytes, which can shape the metabolic and inflammatory microenvironment through released factors and direct cell–cell contacts (58).

### 3.6 Why microglial biology points toward immunometabolism

Although receptor signaling and inflammatory transcription are essential for understanding cholinergic control of microglia, they cannot fully explain the diversity, plasticity and disease specificity of microglial responses. More and more evidences show that these characteristics are rooted in cell metabolism (10, 39). The same cholinergic signal may produce different results, depending on whether microglia are in a glycolytic inflammatory state, a mitochondrial stress state, or a more stable oxidative metabolic state (59). Similarly, the transition from acute activation to chronic maladaptive inflammation is usually accompanied by metabolic remodeling, which affects redox balance, phagocytic capacity and tissue toughness (41, 59).

Recent high-impact reviews have consolidated evidence linking microglial metabolic sensors—such as AMPK, mTOR, and HIF-1α—to inflammatory outcomes in neurodegenerative diseases, although direct evidence for cholinergic signaling regulating these sensors in microglia remains largely correlative or inferred from other cell types (59). Therefore, immune metabolism has become the key missing level in the immune regulation model of cholinergic nerve. If microglia are the cellular hub, metabolic reprogramming may be the mechanism that determines how cholinergic signals are decoded and transformed into functional inflammatory output. The next section looks closely at immunometabolism as the connecting link, and it separates known pathways from ideas that need more checking. As will be discussed in Section 4, both aging and sex have profound effects on microglial metabolism, thereby potentially modulating the efficacy of cholinergic interventions—a consideration that should inform future experimental design and trial stratification (44).

## 4 Immunometabolism: the missing link between cholinergic signaling and inflammatory outcomes

**Figure 3:**
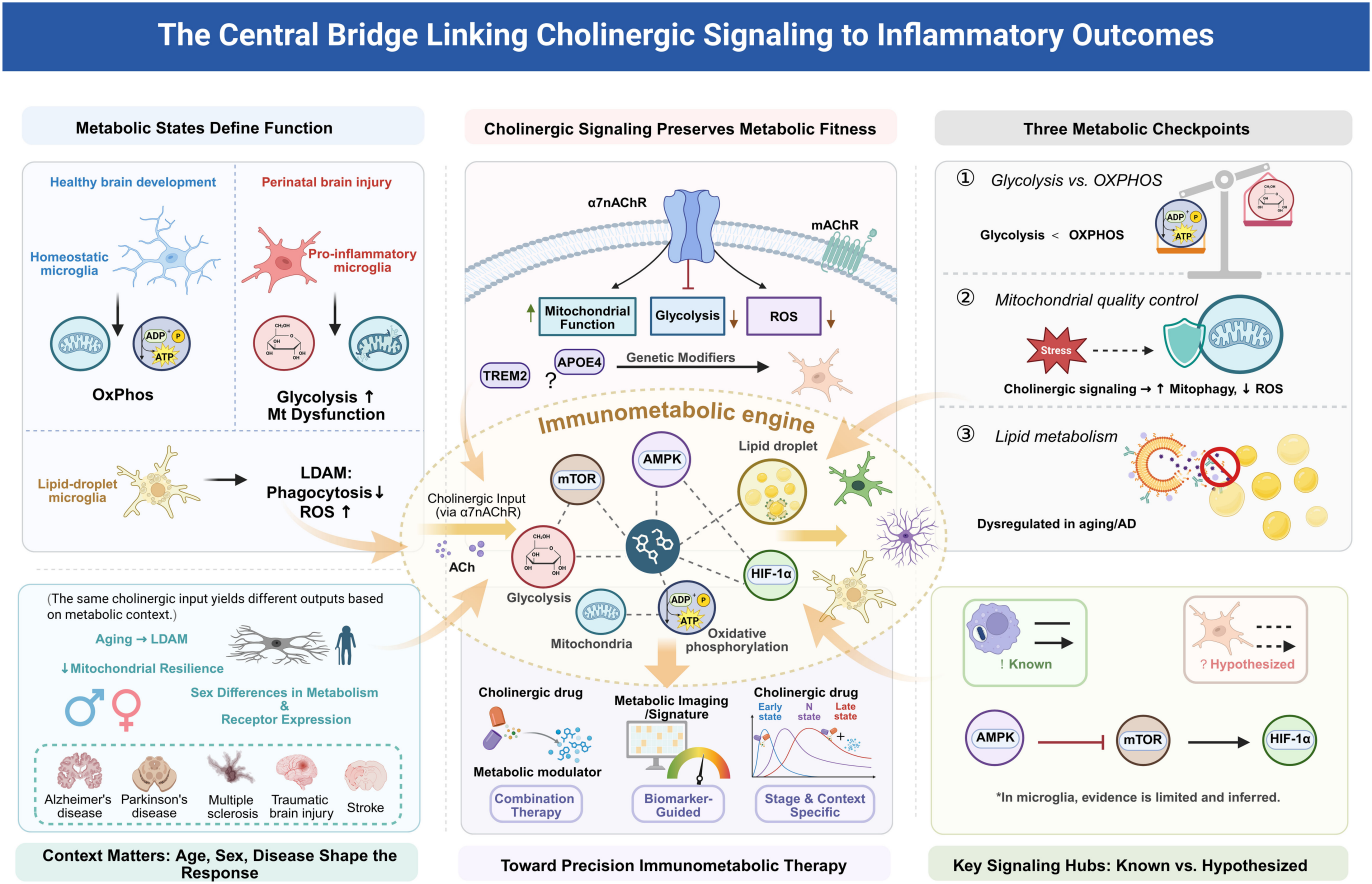
Immunometabolism as the mechanistic bridge between cholinergic signaling and inflammatory outcomes in microglia.

In recent years, immune metabolism has become a core framework for understanding how immune cells adjust their functions according to environmental stress, nutritional availability and inflammatory challenges (60, 61). Cell metabolism is not only a passive energy source, but also actively shapes the phenotype, signal output and survival of immune cells (61). This concept is particularly important in CNS, because microglia are in a highly specialized metabolic environment and must continuously respond to neuronal activity, oxidative stress, tissue damage and age-related steady-state decline. In this context, immune metabolism provides a mechanism bridge between cholinergic signaling and diverse inflammatory outcomes observed in nervous system diseases (**Figure 3**).

### 4.1 Microglial immunometabolism in physiological and inflammatory states

Under steady-state conditions, microglia maintain dynamic metabolic balance to support monitoring function, synaptic maintenance and phagocytic readiness (62). These functions depend on the coordination of oxidative phosphorylation, mitochondrial respiration and flexible substrate utilization (62). However, when microglia are stimulated by inflammation, their metabolic characteristics can change significantly. Similar to peripheral innate immune cells, activated microglia usually show enhanced glycolysis, changes in tricarboxylic acid (TCA) activity, mitochondrial stress and abnormal lipid processing. These changes are not simply secondary to activation, but help to determine the intensity, duration and nature of inflammatory response (63).

Proinflammatory microglial status is usually associated with increased glycolytic flux, accumulation of inflammatory metabolites, and decreased mitochondrial efficiency (64). These metabolic adaptations can provide rapid ATP supply and biosynthetic support for cytokine production, but may also promote reactive oxygen species production, inflammasome activation and persistent tissue damaging inflammation (64, 65). In contrast, the state of microglia that is closer to homeostasis or repair seems to be more dependent on complete mitochondrial function, oxidative metabolism and controlled lipid utilization (65). Although these distinctions are not absolute, they provide a useful framework for understanding how metabolism limits or amplifies the inflammatory potential in the brain.

Lipid droplet-accumulating microglia (LDAM) have recently appeared as a distinct metabolic phenotype with translational relevance. LDAM show impaired phagocytosis, elevated oxidative stress, and dysregulated lipid metabolism, and they are found in aging brains and across many neurodegenerative conditions (66, 67). In Alzheimer’s disease, amyloid-β exposure directly drives LD formation through the enzyme DGAT2, and pharmacological targeting of DGAT2 restores microglial phagocytic function and reduces plaque pathology (68). These observations show that microglial metabolic states are descriptive markers and they are causally linked to functional outcomes.

In signaling, the JAK2-STAT3 pathway has been identified as a key transducer that links α7nAChR activation to metabolic and inflammatory outputs. In macrophages, α7nAChR stimulation recruits JAK2 to the receptor complex, and this causes STAT3 phosphorylation and then suppresses NF-κB-driven pro-inflammatory gene expression (69). Although direct evidence in microglia remains limited, the α7nAChR–JAK2–STAT3 axis may similarly influence metabolic reprogramming by modulating the expression of glycolytic enzymes or mitochondrial regulators. This pathway represents a promising mechanistic link between cholinergic signaling and microglial immunometabolism that warrants further investigation.

### 4.2 Cholinergic signaling as a regulator of microglial metabolic state

More and more evidence shows that cholinergic signal can not only affect the inflammatory behavior of microglia by directly inhibiting cytokine pathway, but also play a role by regulating cell metabolism (70). Under a variety of experimental conditions, the activation of α7nachr and related cholinergic pathways is related to the improvement of mitochondrial integrity, the reduction of oxidative stress and the reduction of inflammatory metabolic reprogramming (71). These observations suggest that cholinergic input may help maintain or restore the metabolic state that is not conducive to the amplification of chronic inflammation.

One possible mechanism is that cholinergic signaling limits the pathological glycolytic transition common in pro-inflammatory activation. In many immune cells, over dependence on glycolysis is associated with inflammatory transcription programs, activation of inflammasomes, and decreased ability to resolve inflammation (72). If cholinergic signaling can inhibit this transition, it may reduce the metabolic support required for sustained cytokine production. Meanwhile, cholinergic stimulation may promote mitochondrial health, reduce reactive oxygen species from mitochondria, and maintain oxidative metabolism, thus supporting a more regulated inflammatory phenotype (72, 73). Although the specific extent of this metabolic effect in microglia is not completely clear, the existing evidence strongly supports that the role of cholinergic signaling is not limited to the inflammatory transcriptional regulation at the proximal end of the receptor.

The intersection between cholinergic signaling and microglial metabolism may also involve genetic risk factors such as TREM2 and APOE. TREM2 is a main controller of microglial energy health, and its level is directly related to sugar uptake and total energy capacity in microglia, in any disease (74). APOE4, the strongest genetic risk factor for late-onset Alzheimer’s disease, harms fat balance in microglia and causes an inflammation-related metabolic state (44). Importantly, cholinergic signals have been shown to affect microglial responses in a way that depends on the APOE type (60). It is still unknown if cholinergic stimulation can change TREM2 expression or function, or fix the metabolic problems linked to APOE4. This needs careful study.

### 4.3 Key metabolic pathways linking cholinergic signaling to inflammatory phenotype

To understand the control of cholinergic on neuroinflammation, several metabolic checkpoints are particularly important. The first is the balance between glycolysis and oxidative phosphorylation. In the process of acute inflammatory activation, microglia usually enhance glycolytic flux, while mitochondrial efficiency decreases (64, 75). This glycolytic redirection is associated with rapid cytokine synthesis and persistent inflammation. Cholinergic signaling may offset this trend by maintaining mitochondrial respiration and reducing the demand for inflammatory glycolysis compensation (71, 73).

The second important axis is mitochondrial quality control. Mitochondria are not only energy producing organelles, but also important regulators of ROS production, calcium homeostasis, apoptosis and innate immune signals (76). Mitochondrial dysfunction can activate the redox sensitive inflammatory pathway and promote the activation of NLRP3 inflammasome, thereby strengthening the maladaptive microglial state (76). In the inflammatory model, cholinergic stimulation is related to the reduction of mitochondrial stress and the improvement of redox balance (77), suggesting that part of its anti-inflammatory effect may come from the stability of mitochondrial function, not just the inhibition of inflammatory gene expression.

The third metabolic checkpoint involves lipid metabolism. Microglia rely on strictly regulated lipid uptake, storage and oxidation to complete membrane remodeling, phagocytosis and injury response (78). Lipid metabolic disorders are increasingly considered to be associated with dysfunctional microglial status in aging and neurodegeneration, including lipid droplet accumulation and impaired phagocytosis (78, 79). Although the relationship between cholinergic signaling and lipid processing in microglia is still poorly studied, it may be an important direction for future research, especially in Alzheimer’s disease, multiple sclerosis and other diseases with closely related lipid metabolism and pathological process (79).

### 4.4 Signaling hubs that integrate cholinergic input and metabolic output

The metabolic effects of cholinergic signaling may be mediated by a group of intracellular signaling hubs, which coordinate energy perception, inflammatory transcription and stress response. Among these, AMP-activated protein kinase (AMPK), mammalian target of rapamycin (mTOR), and hypoxia-inducible factor 1-alpha (HIF-1α) have emerged as central integrators of immune and metabolic cues. AMPK acts as a cellular energy sensor; its activation generally suppresses glycolysis and promotes catabolic, energy-conserving responses, and has been associated with anti-inflammatory outcomes in microglia. mTOR integrates nutrient availability and growth signals; hyperactivation of mTOR supports inflammatory biosynthesis and inhibits autophagic clearance. HIF-1 α is a key transcriptional regulator. It can start the glycolytic conversion process and promote the expression of pro-inflammatory cytokines under inflammatory conditions (80).

Cholinergic signaling may act mainly through the α7 nicotinic acetylcholine receptor (α7nAChR). It could engage one or more of these molecular pathways. This engagement would help microglia maintain a state of metabolic resilience. In peripheral macrophages, activation of α7nAChR increases the phosphorylation of AMPK. It also suppresses the mTOR pathway. Under low oxygen or inflammatory conditions, this same stimulation reduces the stabilization of HIF-1α. However, we still lack direct evidence for these effects in microglia. Researchers do not yet know whether cholinergic stimulation modulates AMPK, mTOR, or HIF-1α specifically in these cells. This knowledge gap becomes even more obvious in the context of neurological disease or aging. Most of our current understanding comes from studies that used peripheral immune cells. It also comes from work on non-microglial brain cells or on microglial cell lines. Those experiments were often conducted under non-physiological conditions (81).

The conceptual framework that links cholinergic input to microglial metabolism through AMPK, mTOR, and HIF-1α is compelling. But when it comes to microglia, this idea remains largely hypothetical. We need to make a critical distinction here. We must separate what we know from direct experiments in microglia from what we infer from other cell types or systems. This distinction is not just an academic exercise. It has real consequences for drug development. Therapies designed to target these metabolic hubs might fail. They could fail if the assumed pathway does not actually operate in microglia under disease-relevant conditions.

Systematic validation using physiologically relevant models—such as primary microglia from aged or disease-state animals, or *in vivo* metabolic imaging—is urgently needed.

### 4.5 Immunometabolism as an explanation for disease-specific variability

A major challenge in this field is the difference of cholinergic effect in different disease models and clinical states. In some cases, cholinergic stimulation can significantly reduce inflammatory injury; In other cases, the benefits may be small, short-lived or difficult to repeat. Immune metabolism may help explain this heterogeneity. The same cholinergic input is unlikely to have exactly the same effect in young steady-state brain, aging brain with metabolic pressure, ischemic lesions or neurodegenerative environment rich in protein pathology (82). In each context, microglia enter different metabolic states, which may change receptor signaling, downstream pathway participation and inflammatory plasticity.

For example, aging is associated with mitochondrial decline, lipid metabolism changes, oxidation-reduction imbalance and chronic low-grade inflammatory preconditioning in microglia (83). These age−related changes—including the emergence of lipid droplet−accumulating microglia (LDAM) and reduced mitochondrial resilience—may substantially alter the responsiveness of aged microglia to cholinergic anti−inflammatory signals or shift the balance of their downstream effects (66).

Sex differences are another critical biological variable. But they are still not studied enough. Female microglia have transcriptional and metabolic profiles that differ from those of male microglia. Researchers have also reported sex differences in microglial cholinergic receptor expression in several disease contexts (44). For example, in models of Alzheimer’s disease, female microglia show stronger inflammatory priming. They also display different metabolic responses to immune challenges. These differences may affect how sensitive they are to cholinergic modulation (84). So, we need to take sex into account as a biological variable. This step is essential when we interpret preclinical data. It is also essential when we design clinical trials of cholinergic interventions that are stratified by sex.

Similarly, in neurodegenerative diseases, the presence of chronic protein aggregation, synaptic stress and sustained glial activation may form a metabolic environment significantly different from acute inflammatory injury (85). This means that cholinergic intervention should not be regarded as a universal equivalent strategy, but should be understood as a background dependent regulatory means, and its efficacy depends on the metabolism and inflammatory state of the target tissue.

### 4.6 From mechanistic insight to therapeutic opportunity

First, it changes the therapeutic concept of cholinergic immune regulation from a method that only focuses on receptors to a way of thinking that combines cellular state and metabolic context. Secondly, it shows that the method of combining cholinergic regulation with metabolic intervention may be superior to a single method. Finally, it also suggests that metabolic signatures can be used to identify new biomarkers of therapeutic responsiveness, such as mitochondrial metabolism or inflammation related metabolism (26). These methods will become particularly important when dealing with neurological diseases, because treatment timing, disease stage, and biological heterogeneity often determine clinical outcomes.

From a broader perspective, integrating cholinergic signaling with microglial immune metabolism can more comprehensively explain how neural activity affects inflammation in a disease - and tissue-specific context. Compared with the classical anti-inflammatory reflex model, this theory is not only more abundant in mechanism, but also more consistent with the complexity involved in neurological diseases. The next challenge is to explore how the above mechanisms can be translated into common neurological diseases, in which cholinergic dysfunction, microglial activation and metabolic dysfunction often occur simultaneously.

## 5 Disease relevance in neurology

When analyzed in connection with different diseases of the nervous system, the relationship between cholinergic nerve regulation and immunity is very obvious. In many diseases, cholinergic dysfunction, microglial activation, and metabolic problems are interrelated events, which may affect the occurrence, progression, and even rehabilitation of the disease (86). Although the nature and extent of interactions may differ in different diseases, there seems to be a potential common thread. It is generally seen that when there is any loss or disturbance of cholinergic signaling, a maladaptive neuroinflammatory state will ensue, and the metabolic response of microglia may determine whether inflammation is protective or develops into a persistent state (27). This section highlights how these themes converge in major neurological disorders (**Table 1**).

**Table 1:** Disease relevance of cholinergic neuroimmune regulation in neurology.

Disease context	Core relevance	Potential value	Key caveat
Alzheimer’s disease and brain aging	Cholinergic loss overlaps with persistent microglial activation and metabolic decline.	May help lower inflammatory tone beyond symptomatic treatment.	Aging may weaken response; clinical benefit is still uncertain.
Parkinson’s disease and synucleinopathies	Cholinergic dysfunction coexists with alpha-synuclein-driven inflammation and oxidative stress.	alpha7nAChR targeting may reduce innate immune injury.	Effects likely vary by stage and central-peripheral context.
Stroke and post-stroke neuroinflammation	Ischemia triggers acute glial activation, cytokine release, and autonomic imbalance.	Timed cholinergic stimulation may limit injury and support recovery.	Over-suppression could impair repair if used too late or too long.
Multiple sclerosis and autoimmune neuroinflammation	Cholinergic pathways intersect with glial activation, BBB dysfunction, and remyelination.	Could reduce inflammation while supporting repair-oriented glia.	Evidence remains limited across lesion and immune states.
Delirium and perioperative brain dysfunction	Systemic inflammation can prime microglia and disturb BBB and network stability.	Useful setting for short-window translational testing.	Drug benefits are mixed despite strong mechanistic rationale.
Cross-disease perspective	Cholinergic dysfunction often converges with maladaptive microglial states.	Supports a shared neuroimmune axis for patient stratification.	Outcome depends on timing, tissue context, and baseline state.

BBB, blood-brain barrier; alpha7nAChR, alpha7 nicotinic acetylcholine receptor.

### 5.1 Alzheimer’s disease and brain aging

Loss of cholinergic cells in the basal forebrain is a feature of the disease (87). This phenomenon is traditionally considered to be the cause of cognitive dysfunction and constitutes the basis for the application of cholinesterase inhibitors (71). At the same time, more and more studies have shown that cholinergic defects may also lead to changes in the balance of neuroinflammation in older patients. With progressive cell death, ad shows signs of persistent microglial activation, abnormal synaptic pruning, inflammation caused by amyloid deposition, and metabolic stress (45, 63, 71), These findings suggest that the cholinergic defect associated with AD is not just a neurotransmitter deficiency.

In AD, microglia show a high degree of heterogeneity. This heterogeneity includes disease-associated microglia (DAM), lipid-droplet-accumulating microglia (LDAM), and other subtypes defined by their metabolic state. These phenotypes emerge in response to amyloid plaques, tau pathology, and tissue stress (63, 66, 68), TREM2 is a key regulator of microglial metabolic fitness. It is required for the transition to DAM states. Its expression levels correlate with glucose uptake, phagocytic capacity, and overall immunometabolic plasticity (60, 74). TREM2 also mediates how microglia respond to lipid imbalance. It modulates cholesterol metabolism and in this way prevents harmful lipid accumulation (88). By contrast, APOE4 is the strongest genetic risk factor for late-onset AD. It disrupts lipid balance in microglia and promotes a pro-inflammatory metabolic state. It also reshapes the protein makeup of lipid droplets. These changes alter microglial inflammatory responses (89). Of note, exposure to amyloid-β directly causes lipid droplets to form. This process depends on the enzyme DGAT2. Inhibiting DGAT2 helps microglia recover their phagocytic function. It also reduces amyloid plaque pathology (68). These lipid-related phenotypes are more than just descriptive markers. They have a causal link to functional outcomes. These outcomes include impaired phagocytosis, elevated oxidative stress, and chronic inflammatory signaling. All of these directly impact the progression of AD.

Cholinergic signaling interacts with these risk-associated pathways. This interaction adds another layer of complexity. Emerging evidence suggests that cholinergic signals may change TREM2 expression or function in microglia. Yet, the exact mechanisms are still not known.APOE4 has been shown to alter how microglia respond to cholinergic input. It also changes the expression of cholinergic receptors. One example is the α9 nicotinic acetylcholine receptor (α9 nAChR) subunit. This receptor has been linked to the regulation of microglial inflammatory responses in the setting of amyloid pathology (90). The α9 nAChR subtype often forms heteromeric receptors together with the α10 subunit. Recent work has shown that it mediates the anti-inflammatory effects of cholinergic signaling in microglia. It may represent a therapeutic target in AD that has not been fully explored. Together, these observations point to a bidirectional interplay. This interplay involves APOE4 carrier status, the lipid metabolic state of microglia, and the cells’ sensitivity to cholinergic signaling. It needs systematic investigation. This metabolic and genetic heterogeneity likely helps explain why AD patients show variable responses to cholinergic interventions.

The possible cholinergic regulation of the above reactions may be achieved through restraining the production of excessive cytokines, decreasing inflammasome activation, and maintaining the regulatory ability of microglia (75). Under such circumstances, some physiological actions exerted by acetylcholinesterase inhibitors can be attributed to the increase in ACh neurotransmission as well as the modulation of inflammation (91). Despite the uncertainty regarding the anti-inflammatory roles played by the agents used in the therapy of AD, the physiological basis appears to be increasingly reasonable.

Astrocytes also participate in cholinergic neuroimmune regulation in AD. When faced with amyloid pathology, astrocytes undergo reactive changes, which may exacerbate neuroinflammation or support neuronal survival depending on the specific context and disease stage. Astrocytes express multiple cholinergic receptor subtypes, including α7nAChR and muscarinic M1 and M3 receptors, and cholinergic signaling has been shown to influence astrocytic inflammatory gene expression, Ca²^+^ signaling, metabolic support to neurons, and glial scar formation (30, 58). Notably, in AD mouse models, activation of the M1 mAChR reduces both microglial and astrocytic activation, supporting the concept that cholinergic modulation of neuroinflammation involves coordinated effects on both glial populations (20). The potential role of astrocytes in cholinergic neuroimmune regulation in Alzheimer’s disease deserves further investigation, especially considering the significant metabolic changes observed in astrocytes in this disease.

Aging can worsen the interaction even more. In aged brains, microglial cells usually get more reactive, metabolically deprived, and become less efficient at regulating homeostasis (83). This might alter the microglia reaction to cholinergic stimulation, or alter the ratio of the latter effects. Thus, Alzheimer’s disease can be considered the result of the combined influence of decreased cholinergic transmission, poor adaptation of microglia, and immune metabolism disorder; this is an excellent example of studying failure of the cholinergic-immune system communication under chronic neurological stress (91).

Sex differences also deserve attention in Alzheimer’s disease. Female sex is a major risk factor for AD. Female microglia show metabolic and inflammatory profiles that are distinct from those of male microglia. These differences include altered expression of cholinergic receptors and changed responses to amyloid pathology (44, 92). However, we still do not know whether these sex-dependent differences affect the efficacy of cholinergic interventions in AD. Existing preclinical studies often fail to include both sexes. They also rarely analyze data separately by sex. This gap limits the generalizability of the current conclusions. Future studies should therefore treat sex as a biological variable. Researchers should do this when they evaluate cholinergic neuroimmune modulation in AD models.

### 5.2 Parkinson’s disease and related synucleinopathies

Parkinson’s disease (PD) and related synuclein diseases also involve the complex interaction between cholinergic signals and neuroinflammation (93). Although PD is classically defined by dopaminergic degeneration, cholinergic dysfunction is increasingly recognized as an important contributor to motor and non-motor symptoms, including cognitive impairment, gait disorders, autonomic nerve disorders, and sleep disorders (93, 94). At the same time, the activation of microglia, inflammatory cytokine signaling and α-synuclein-drive innate immune activation are continuously considered to be involved in the pathogenesis of PD (95).

Microglia respond to misfolded or aggregated α-synuclein by forming inflammation and stress-related States, which may promote neuronal vulnerability (96). These responses are affected by mitochondrial dysfunction and oxidative stress, which are the core characteristics of PD biology (96). Because cholinergic signal is involved in autonomic nerve regulation, basal forebrain function and striatal loop, its destruction may lead to abnormal regulation of central and peripheral inflammation. Experimental evidence suggests that cholinergic pathway, especially through α 7nachr, may weaken the inflammatory response of microglia to pathological stimulation and reduce oxidative damage. Genetic background also plays a modifying role: the APOE4 allele has been shown to reshape the microglial lipid droplet proteome and modulate inflammatory responses, which could influence the sensitivity of microglial cells to cholinergic anti-inflammatory signals in PD (89).Although the evidence of transformation is still limited, these findings support that cholinergic neuroimmune regulation is not only related to the treatment of symptoms, but also may be related to PD disease modification strategies.

Another factor to be considered in PD is significant autonomic nerve dysfunction. Vagal and parasympathetic nerve abnormalities are common, and may occur before motor performance in some patients (97). This raises the possibility that peripheral cholinergic signal changes and central nervous system inflammation may interact on a longer time scale. This interaction may be particularly important in the progression of prodromal or non-motor symptoms. At this time, bioelectronics or targeted cholinergic intervention has theoretical value (98).

Astrocytes may also contribute to cholinergic neuroimmune interactions in PD. α-Synuclein pathology induces reactive astrocytosis, and astrocytic cholinergic receptor expression—particularly α7nAChR—has been linked to the regulation of inflammatory and oxidative stress responses. Moreover, astrocytes play a central role in brain metabolic homeostasis and can modulate microglial function through the release of metabolites and trophic factors, suggesting that cholinergic signaling may exert immune-regulatory actions that involve both glial populations (30, 58).

Age and sex also modulate the relationship between cholinergic function and neuroinflammation in PD. Aging is associated with progressive loss of cholinergic neurons in the basal forebrain and brainstem cholinergic nuclei, as well as with age-related changes in microglial metabolism that may blunt the responsiveness of microglial cells to cholinergic input. Sex differences have also been recognized: the incidence and clinical presentation of PD differ between males and females, and sex-dependent differences in microglial cholinergic receptor expression and metabolic profiles have been reported (44). A more precise understanding of how age- and sex-related factors influence cholinergic neuroimmune signaling in PD is needed to guide patient stratification in future interventional trials.

### 5.3 Stroke and post-stroke neuroinflammation

Stroke represents another equally important background, in which cholinergic signals, microglia activation and immune metabolic changes converge (98, 99). After acute ischemic injury, rapid aseptic inflammatory response occurs in the brain, involving resident glial cells, infiltrating leukocytes, endothelial activation and cytokine release (99). Microglia are one of the earliest responders and play a dual role: they are involved in cell debris clearance and repair initiation, but they may also amplify secondary injury through inflammatory mediators and oxidative stress (100). The balance between protective and detrimental microglial responses is highly dependent on disease stage, tissue context, and the metabolic state of the cells—a key consideration when designing stage-specific cholinergic interventions. At the same time, stroke is also accompanied by significant disturbance of autonomic nerve balance and systemic immunity, including inflammatory activation and post-stroke immunosuppression (100).

Cholinergic pathway may regulate stroke pathology at multiple levels. At the central level, they may affect the phenotype of microglia, inflammatory transmission and neuronal vulnerability (38). At the peripheral level, vagal and autonomic mechanisms may shape the systemic immune response, thereby affecting the risk of infection, recovery and long-term outcome. Experimental studies suggest that cholinergic stimulation or α 7nachr activation can reduce infarction related inflammation, reduce cytokine production, and improve functional recovery in some models (101). Mechanistically, activation of microglial α7nAChR has been linked to the JAK2-STAT3 signaling pathway, which plays a critical role in modulating glycolytic metabolism and inflammatory cytokine production in microglia after ischemic injury (69). α7nAChR stimulation may also influence metabolic reprogramming by regulating AMPK-mTOR-associated autophagy and preserving mitochondrial integrity. These observations indicate that cholinergic signaling exerts immunometabolic control over microglial responses in the ischemic brain, with implications for the timing and targeting of VNS-based therapies.

In addition to microglia, astrocytes contribute to cholinergic modulation of post-stroke inflammation. Astrocytes are critical for maintaining blood–brain barrier integrity, regulating glutamate homeostasis, and providing metabolic support to neurons. After ischemic injury, cholinergic signaling in astrocytes can influence reactive astrogliosis, glial scar formation, and the release of neurotrophic or inflammatory factors (58). Notably, the α7nAChR in astrocytes has been shown to mediate anti-inflammatory and antioxidant effects in stroke models, suggesting that both microglial and astrocytic cholinergic responses should be considered when designing neuroimmune interventions for stroke (102).

However, stroke also highlights the importance of timing and disease stage. In the acute phase, inhibition of inflammation may reduce tissue damage; In the subacute phase or recovery phase, a certain degree of microglia activation is necessary for repair and remodeling (103). Therefore, it cannot be assumed that cholinergic intervention in stroke has a consistent benefit at all time points. The challenge is not only to activate anti-inflammatory signals, but also to achieve this in a way that retains beneficial clearance and repair functions while preventing excessive activation of inflammation (104).

Aging makes the assessment of cholinergic interventions in stroke more complicated. Aged microglia have a higher baseline inflammatory tone. They also show impaired mitochondrial function and reduced metabolic plasticity. These features may make them less responsive to cholinergic signals and may also shift the balance of downstream effects (105). These age-related differences point to a clear need. Researchers should use preclinical models that reflect an aged system. Stroke trials that evaluate cholinergic neuromodulation should also carefully stratify patients by age. This makes stroke a particularly useful model for studying the phase dependent effects of cholinergic nerve immune regulation.

### 5.4 Multiple sclerosis and autoimmune neuroinflammation

Multiple sclerosis (MS) and related autoimmune neuroinflammatory diseases provide another framework for the clinical and mechanism relevance of cholinergic regulation (104). In MS, inflammatory demyelination results from the interaction between peripheral immune infiltration, CNS resident glial cells, blood-brain barrier dysfunction and repair failure (106). Although adaptive immunity has long been emphasized as the core of MS pathogenesis, microglia and macrophages are now considered to play a key role in lesion expansion and remyelination (106). Cholinergic signals may affect this environment at many levels. At the central level, cholinergic regulation of microglia may affect inflammatory amplification and tissue damage induced innate immune response. At the peripheral level, autonomic and vagal pathways may alter systemic immune activation and immune cell migration. The experimental model of autoimmune neuroinflammation suggests that the α 7nachr related pathway can reduce the severity of inflammation and may affect the balance of cytokines and the behavior of innate immune cells (107). Beyond α7nAChR, both muscarinic receptors and non-α7 nicotinic receptor subtypes (e.g., α4β2, α9-containing nAChRs) also contribute to the modulation of neuroinflammation in MS models. Activation of mAChRs has been shown to attenuate microglial pro-inflammatory phenotypes, and nicotinic receptors such as α9 nAChR have been shown to differentially regulate autoimmune neuroinflammation in experimental autoimmune encephalomyelitis (EAE, 22, 108). The recent demonstration of α9 nAChR expression in human microglia further supports its potential relevance to MS pathophysiology.

In addition, microglial metabolism is increasingly considered to be an important factor in determining whether glial activation supports remyelination or promotes the activity of chronic lesions (109). Lipid droplet-accumulating microglia (LDAM), which have been identified in MS lesions, exhibit impaired phagocytosis and dysregulated lipid metabolism—features that may contribute to failed remyelination and chronic lesion activity (43, 66). The potential for cholinergic signaling to modulate LDAM formation or function in the MS context remains to be explored but represents a promising direction for future research. This raises the possibility that cholinergic signaling is not only related to the inhibition of inflammation, but also may promote recovery by affecting the metabolism and function of glial cells.

Although the definitive clinical evidence is still limited, Ms illustrates an important principle: cholinergic nerve immune regulation may not only be limited to the inhibition of innate inflammation, but also include the effects on repair, cell crosstalk and lesion evolution. This broader perspective may be particularly useful for diseases with focus on inflammation control and tissue regeneration (109).

### 5.5 Delirium, perioperative neurocognitive disorders, and systemic inflammation-associated brain dysfunction

Acute brain dysfunction associated with systemic inflammation, such as delirium, sepsis related encephalopathy and perioperative neurocognitive impairment, may be particularly related to cholinergic neuroimmune regulation (110). “Cholinergic deficiency hypothesis” has long been used to explain delirium, which is based on the importance of cholinergic signals in attention, arousal and cognitive integration (110). At the same time, systemic inflammation can activate immune pathways in the brain, change the permeability of blood-brain barrier, pre stimulate microglia and destroy the balance of neurotransmitters. Recent studies have highlighted that systemic inflammation-induced microglial metabolic changes—including glycolytic reprogramming, mitochondrial dysfunction, and lipid dysregulation—are critical determinants of the vulnerability to delirium and may interact with cholinergic signaling pathways to modulate disease risk and severity (111, 112).

During acute systemic stress, microglia are highly sensitive to peripheral inflammatory signals (113). In susceptible individuals, including the elderly and patients with underlying neurodegenerative changes, this may lead to exaggerated neuroinflammatory response, mitochondrial dysfunction and brain dysfunction at the acute network level. In this context, cholinergic signaling may play a role as a protective regulatory axis by limiting inflammatory escalation and maintaining network stability. Although the clinical data on the benefits of cholinergic drugs in the prevention or treatment of delirium are still inconsistent, the mechanism relationship between cholinergic signals, neuroinflammation and acute cognitive impairment is still highly reasonable (114). The relationship among cholinergic signaling, neuroinflammation, and acute cognitive dysfunction is highly context-dependent and influenced by age, baseline microglial metabolic state, and the presence of chronic neurodegenerative pathology. Older adults and patients with baseline neurodegenerative changes may exhibit heightened susceptibility to inflammation-induced delirium due to age-related declines in cholinergic tone, pre-existing microglial priming, and reduced metabolic plasticity (115). These observations suggest that both patient age and metabolic-inflammatory status should be considered when designing cholinergic interventions for delirium prevention.

From the perspective of transformation, this disease background is particularly attractive, because the inflammatory triggers can usually be identified, the course of disease is relatively clear, and nerve regulation intervention may be easier to test in the perioperative period or intensive care environment (116). It also emphasizes that neuroimmune regulation should be considered not only in chronic neurodegeneration, but also in acute and reversible brain dysfunction.

### 5.6 Shared themes across neurological diseases

Although these diseases have significant differences in etiology, time scale and pathology, they reveal several common principles. First, cholinergic dysfunction often coexists with neuroinflammation, which may be either a contributing factor, a downstream result, or both (116). Secondly, microglia phenotype seems to be the key factor determining whether inflammation remains adaptive or becomes destructive (117). Third, the immune metabolic state may explain why similar cholinergic inputs have different effects in aging, neurodegeneration, ischemia, autoimmunity and acute systemic inflammation (117).

Building on these principles, a more integrated framework emerges for understanding cholinergic neuroimmune regulation across diseases. The metabolic and inflammatory state of microglia—including the balance between oxidative phosphorylation and glycolysis, mitochondrial integrity, LDAM burden, and the functional status of key regulatory pathways such as JAK2-STAT3 and AMPK–mTOR–HIF-1α—critically determines the responsiveness of microglial cells to cholinergic signals. Genetic factors such as TREM2 and APOE4 have a profound influence on microglial immunometabolism. They also shape how sensitive microglia are to cholinergic modulation. This influence likely contributes to the heterogeneity in therapeutic outcomes. This heterogeneity can appear across disease stages and among individuals. Age- and sex-dependent changes in cholinergic tone and microglial function add another layer of modulation. They further affect the efficacy of cholinergic interventions. These changes make it clear that we need stratified approaches. Other cholinergic receptor subtypes also play important roles in neuroimmune regulation. These include non-α7 nicotinic receptors, such as α4β2 and α9, as well as muscarinic receptors. Their roles depend heavily on the specific context. Astrocytes are active participants in cholinergic neuroimmune regulation. They influence metabolic support, neuronal survival, and the inflammatory microenvironment. Finally, sex-specific differences in lipid metabolism, immune signaling, and cholinergic tone carry profound implications. They matter for how we stratify patients in trials and for how we design those trials. Therefore, both preclinical models and clinical studies should take these differences systematically into account.

However, all of these considerations make us realize that regulation of immunity by cholinergic nerves should not be seen in terms of a disease-related phenomenon, but rather, as an axis of regulation across diseases in neurology (118). Hence, the difficulty in treatment is not only in activating the cholinergic anti-inflammatory pathway but in defining when, where and on which population regulation by cholinergic nerves would have beneficial effects on the inflammation process. This leads us to the subsequent issue, i.e., neural regulation and medication interventions.

## 6 Neuromodulation and therapeutic translation

The transformation attraction of cholinergic nerve immune regulation is that the inflammatory response may be regulated by targeted drugs and nerve regulation intervention, rather than relying solely on extensive immunosuppression (118, 119). This is particularly important in neurology, because chronic or excessive inflammation often coexists with fragile tissue structure, limited regeneration ability and significant disease heterogeneity (119). In this case, treatment that can recalibrate rather than completely eliminate inflammatory signals may provide a more favorable balance between efficacy and safety (108). The cholinergic system is well suited to support this therapeutic logic because it connects nerve activity, autonomic nerve regulation, glial reactivity, and immune cell function in the central and peripheral compartments (120) (**Table 2**).

**Table 2:** Translational strategies for cholinergic neuroimmune regulation in neurology.

Translational domain	Main strategy	Potential benefit	Key challenge/consideration
Acetylcholinesterase inhibitors	Enhance endogenous cholinergic tone by increasing synaptic acetylcholine availability.	Clinically established in neurology; may modulate neuroinflammation, glial activation, and neurovascular homeostasis in addition to symptomatic cognitive effects.	Anti-inflammatory benefit is difficult to separate from symptomatic improvement; effects likely vary by disease and stage.
Selective nicotinic receptor targeting (especially alpha7nAChR)	Use agonists or partial agonists to engage anti-inflammatory cholinergic signaling with greater receptor specificity.	Offers a more mechanistically focused strategy and may reduce inflammatory signaling in microglia and immune cells without broad cholinergic stimulation.	Receptor desensitization, off-target effects, variable receptor expression, and unclear balance of central versus peripheral actions may limit translation.
Muscarinic pathway modulation	Target muscarinic receptors to influence CNS neuromodulation, astrocyte behavior, immune-cell responses, and neurovascular function.	Represents an underexplored therapeutic space that could widen cholinergic control beyond nicotinic pathways.	Evidence for clinically meaningful anti-neuroinflammatory effects remains limited and mechanistic specificity is still uncertain.
Vagus nerve stimulation (VNS)	Implanted bioelectronic neuromodulation engaging autonomic tone, inflammatory reflex pathways, and brainstem-integrated neuroimmune regulation.	May simultaneously affect central networks, peripheral immune signaling, cytokine production, microglial activation, mitochondrial resilience, and tissue injury.	Clinical translation is incomplete because stimulation parameters vary, disease-specific targets remain unclear, and reliable engagement biomarkers are still lacking.
Non-invasive neuromodulation (for example taVNS)	Use transcutaneous or other non-invasive stimulation methods to modulate vagal-autonomic inflammatory pathways without surgery.	More accessible and safer than implanted systems; useful for exploratory testing in stroke recovery, pain, epilepsy, depression, cognitive impairment, and perioperative brain dysfunction.	Current evidence is still relatively small-scale or exploratory, and optimal protocols and responsive patient groups are not yet established.
Biomarker-guided precision translation	Combine autonomic, glial, inflammatory, imaging, transcriptomic, and metabolomic readouts to identify tractable biology and target engagement.	Could improve patient stratification, reveal cholinergic tone and microglial state, and move the field toward biologically guided intervention.	Robust, disease-relevant biomarkers are not yet standardized; common surrogates such as heart rate variability have limited specificity for cholinergic anti-inflammatory signaling.
Stage- and context-dependent therapeutic design	Match cholinergic intervention to disease type, biological state, and timing rather than applying a uniform anti-inflammatory model.	Supports precision neuroimmune therapy in which the goal is to recalibrate inflammatory set points instead of broadly suppressing immunity.	Benefits may reverse with disease stage; interventions helpful in acute injury or early neurodegeneration may become ineffective or counterproductive later.

ACh, acetylcholine; VNS, vagus nerve stimulation; taVNS, transcutaneous auricular vagus nerve stimulation; CNS, central nervous system.

### 6.1 Pharmacological strategies targeting cholinergic neuroimmune pathways

A direct method of transformation is to enhance cholinergic signals through drugs. The most mature drugs in neurology are acetylcholinesterase inhibitors, which can increase the availability of acetylcholine in synapses and are widely used in Alzheimer’s disease and related cognitive impairment (120). Although these drugs were originally developed to improve neurotransmission, their broader biological effects may include the regulation of neuroinflammation, glial cell activation and neurovascular response (91, 121). Experimental studies suggest that enhancing endogenous acetylcholine can reduce inflammatory signals and improve tissue homeostasis in some cases (121). However, its clinical anti-inflammatory effect is still difficult to distinguish from cognitive or symptom improvement effects, and the magnitude of this effect may be significantly different between different diseases and disease stages (122).Clinical evidence for the anti-inflammatory effects of cholinesterase inhibitors in neurological disorders remains limited and inconsistent. In Alzheimer’s disease, researchers have conducted several randomized controlled trials. These trials tested the effects of donepezil or rivastigmine on inflammatory biomarkers. But the results have been mixed. Some studies reported reductions in pro-inflammatory cytokines. These cytokines, such as IL-1β and TNF-α, were measured in plasma or cerebrospinal fluid. Other studies found no significant changes (123). A meta-analysis drew a conclusion from these data. The anti-inflammatory effects of cholinesterase inhibitors are modest, if they exist at all. They also vary widely from person to person. This variability may come from differences in disease stage. It may also come from differences in baseline cholinergic tone or from other medications patients are taking (124). For conditions like delirium or perioperative cognitive dysfunction, researchers have also run prospective trials. These trials tested donepezil or rivastigmine. Despite a strong mechanistic rationale, they have largely failed to show consistent preventive or therapeutic benefits (125). These observations underscore an important point. Simply increasing the availability of endogenous acetylcholine is unlikely to be enough. It will not achieve robust control of neuroinflammation by itself. Patient selection and the timing of the intervention are equally critical.

Another major drug strategy is to directly target nicotinic receptors, especially α7nAChR (122). Because α7nAChR is closely related to anti-inflammatory signals in peripheral immune cells and microglia, selective agonists and partial agonists have attracted extensive attention as candidate therapies for neuroinflammation and neurodegenerative diseases. The theoretical advantages of this method include higher mechanism specificity and the ability to distinguish anti-inflammatory effects from extensive cholinergic stimulation. However, challenges remain. Receptor desensitization, off target effect, receptor expression differences in different disease states, and insufficient understanding of cell type specificity may limit the success of its transformation (24, 126). In addition, the balance between the central and peripheral effects of these drugs is not always clear, which makes the interpretation of clinical trial results more complicated. Clinical translation of α7nAChR-selective agonists has faced substantial hurdles. Several compounds, including GTS-21 (DMXB-A), MEM3454, and encenicline, have been tested in early-phase trials for cognitive impairment in AD and schizophrenia, but anti-inflammatory outcomes have rarely been included as primary or secondary endpoints (127). In a phase 2 trial of encenicline for cognitive impairment in schizophrenia, no significant improvement was observed, and the compound was subsequently discontinued (128). More recently, a randomized placebo-controlled trial of the α7nAChR positive allosteric modulator AVL-3288 in mild AD found no effect on neuroinflammatory biomarkers (measured by CSF cytokines or PET imaging of microglial activation) despite good target engagement in preclinical models (127). These negative or inconclusive results point to several challenges. One challenge is receptor desensitization. Another is our incomplete understanding of cell-type specificity. This refers to the difference between peripheral and central effects. A further possibility is that α7nAChR activation alone is simply not enough. It cannot overcome neuroinflammation that has already taken hold in late-stage disease. Future trials should use biomarker-based patient selection. For example, they could enroll patients who show signs of elevated microglial activation or reduced cholinergic tone. These trials should also consider combination strategies. Such strategies would target multiple receptors at the same time.

The muscarinic pathway may represent a less explored but potentially important therapeutic field (129). Although muscarinic receptor signaling is classically related to cognition, autonomic nervous control and neural regulation, emerging evidence suggests that it may also affect immune cell behavior, astrocyte activation and neurovascular response (129). Whether muscarinic targeting can produce clinically significant anti-inflammatory effects is uncertain, but given the complexity of cholinergic regulation in CNS, this field deserves more attention (34). To date, no muscarinic receptor-targeting drug has been advanced to phase 2/3 trials specifically for neuroinflammatory indications in neurology. Preclinical studies have tested M1 positive allosteric modulators (PAMs) and observed a reduction in glial activation in mouse models of AD. However, moving these findings into human studies has been difficult. One major obstacle is the occurrence of dose-limiting cholinergic side effects, such as bradycardia and gastrointestinal disturbances. Another is the challenge of achieving sufficient drug penetration into the central nervous system (130). A phase 1 trial examined the M1 PAM VU0486846 in healthy volunteers. This study reported an acceptable safety profile, but it did not include any outcome measures related to neuroinflammation (131). Therefore, targeting muscarinic receptors remains a translational direction that is both underexplored and high in risk. Future progress will require compounds with better selectivity, along with more definitive mechanistic validation under human neuroinflammatory conditions.

### 6.2 Vagus nerve stimulation as a prototype bioelectronic therapy

Among non-drug strategies, VNS has become the most famous transformation application of cholinergic anti-inflammatory concept (34). VNS was initially used in epilepsy and depression, and then was explored for a variety of inflammatory and immune-mediated diseases because it can affect autonomic nerve tension and inflammatory reflex pathways (35). In nervous system diseases, VNS is particularly concerned because it may affect central network function, peripheral immune signals and neuroinflammatory dynamics at the same time (132).

In preclinical studies, VNS is associated with reduced cytokine production, changes in microglia activation, improved mitochondrial toughness, and reduced tissue damage in stroke, neurodegeneration, and systemic inflammation related brain dysfunction models (13, 133). These findings suggest that VNS may not only play a role through the peripheral cap mechanism, but through more complex interactions involving brain stem integration, network regulation and immune regulation (133). However, in clinical practice, the transformation of these effects to the clear benefit of anti-inflammatory neurology is still incomplete. VNS parameters vary greatly, disease-specific targets have not been fully defined, and biomarkers involving targets are still under development (134).

Vagus nerve stimulation (VNS) has been proposed as an anti-neuroinflammatory intervention for neurological diseases. But the clinical evidence for this use is still limited and inconsistent. VNS is already an approved therapy for epilepsy and depression. In these conditions, *post-hoc* analyses have suggested that certain patient subgroups experience decreases in systemic inflammatory markers, such as C-reactive protein and IL-6. However, prospective trials that directly assess neuroinflammatory endpoints are still lacking (135). In the context of stroke, a small randomized trial tested VNS paired with rehabilitation for upper limb recovery. This trial did not report any inflammatory outcomes. It also found no significant difference in functional recovery between the group that received VNS and the sham group (136). VNS has produced more promising results in inflammatory bowel disease, a peripheral inflammatory condition (29). Yet extending these findings to central nervous system disorders is challenging. This challenge is largely due to the presence of the blood–brain barrier and to the degeneration of central cholinergic pathways. These inconsistent clinical findings suggest that VNS may need to be optimized for each disease context with regard to stimulation parameters (frequency, intensity, duty cycle), timing relative to disease stage, and patient selection based on baseline cholinergic function or inflammatory status.

### 6.3 Non-invasive neuromodulation and the expansion of translational possibilities

Due to the high cost and invasiveness of implantable VNS, a large number of researches have turned to non-invasive nerve regulation strategies, especially transcutaneous vagus nerve stimulation (tavns, 134). This method has attracted attention due to its relative safety, good accessibility, and the possibility of regulating autonomic nerve and inflammatory pathways without surgical implantation (137).In neurology, tavns has been studied in the fields of stroke recovery, pain, epilepsy, depression, cognitive impairment and perioperative brain dysfunction (137). Although many studies are still small or in the exploratory stage, this method is attractive because it may provide a practical way to test cholinergic neuroimmune regulation in human diseases.

Other emerging methods include nerve regulation based on ultrasound, electric field stimulation technology, and multimodal bioelectronic systems designed to more accurately regulate the autonomic nerve immune loop (138). These methods are still in the early stage of development, but reflect an important conceptual change: cholinergic nerve immune regulation is no longer only regarded as a drug target, but also as a controllable system level attribute in nerve immune communication. This shift may be particularly important in neurology, because local brain networks, timing of intervention and disease-specific loop vulnerability may determine treatment outcomes.

Despite its promise, clinical evidence for transcutaneous auricular VNS (taVNS) as an anti-neuroinflammatory intervention remains preliminary and inconsistent. In stroke, two small randomized trials reported reductions in serum inflammatory cytokines and improved functional outcomes with taVNS (139), but a larger sham-controlled trial found no significant benefit on either inflammatory markers or disability scores (140). In AD, a pilot study of taVNS (8 weeks) showed improvements in cognitive scales but did not measure neuroinflammatory biomarkers (141), and no sham-controlled trials have been reported. Meta-analyses of taVNS for chronic pain or depression have yielded small effect sizes with high heterogeneity, and publication bias is a concern (142). Furthermore, optimal stimulation parameters (ear location, current intensity, frequency, duration) have not been standardized across studies, making cross-trial comparisons difficult. Thus, while taVNS is a safe and accessible approach, its efficacy for neuroinflammatory conditions is not yet established, and large, well-controlled, biomarker-informed trials are urgently needed.

### 6.4 Biomarkers and patient stratification for precision translation

One of the major obstacles to clinical transformation is the lack of robust biomarkers to identify when cholinergic neuroimmune disorders exist and whether certain interventions really act on relevant biological processes (143).Traditional inflammatory markers, such as circulating cytokines, have limited specificity for CNS inflammation, while neuroimaging and cerebrospinal fluid markers usually only reflect part of the disease process. A more useful transformation strategy may require a combination of biomarkers to reflect autonomic nervous status, inflammatory phenotypes, and possible metabolic or glial status (144).

Heart rate variability has been proposed as a possible alternative indicator of autonomic and vagal function, but its specificity for cholinergic anti-inflammatory signals is limited (145). Other candidate biomarkers, such as plasma acetylcholinesterase activity, serum choline levels, and PET imaging of α7nAChR availability, have been explored in small studies, but none has been validated for predicting response to cholinergic interventions in neurological diseases (146). The dynamic nature of cholinergic receptor expression and the heterogeneity of microglial metabolic states further complicate biomarker development; a single static measurement is unlikely to capture the relevant biology. At the CNS level, glial cell imaging, inflammatory activated humoral markers, and molecular features from transcriptomics or metabonomics may provide more disease-related information (147). In the future, integrating single-cell, spatial, imaging and physiological data sets may achieve more accurate stratification of patients according to microglial status, cholinergic tension and inflammatory metabolic phenotype (147). This method will promote the field from empirical intervention to biological guidance of neural regulation.

### 6.5 Disease stage, timing, and context dependence

An important experience of neuroinflammation research is that the biological significance of inflammation strongly depends on timing and disease background (148). In acute injury, inflammatory response may have protective effect at first, and then become maladaptive. In chronic neurodegeneration, low-grade inflammation may evolve over many years and interact with protein pathology, synaptic dysfunction and aging related metabolic decline (149). These differences have a profound impact on cholinergic therapy. Beneficial interventions in early stroke may be ineffective or even counterproductive if they inhibit the necessary repair process in the recovery period. Similarly, the treatment of improving inflammatory regulation in early neurodegeneration may be of limited value once the structural damage has entered the late stage (149).

This phase dependence is likely to explain some of the inconsistencies observed in preclinical and clinical studies. It also suggests that successful transformation requires a dynamic treatment model rather than a “one size fits all” strategy (150). Furthermore, the effects of cholinergic interventions are modulated by age and sex. Aged microglia exhibit reduced metabolic plasticity and altered cholinergic receptor expression, which may blunt responsiveness to cholinergic stimulation (58, 105). Sex differences in cholinergic tone and microglial immune responses have also been documented; for example, female microglia show greater baseline inflammatory priming and may respond differently to α7nAChR agonists compared to male microglia (44). These variables are rarely accounted for in preclinical studies or clinical trials, potentially contributing to inconsistent outcomes. Therefore, rather than asking whether cholinergic nerve regulation usually has anti-inflammatory effect, the more useful question may be: in which disease, which stage, what biological state, and when there is evidence of measurable target participation, is cholinergic intervention beneficial?

### 6.6 Current limitations and translational challenges

Although significant progress has been made, there are still several challenges that limit the development of therapeutic strategies for cholinergic neuroimmunity. First, many mechanism studies are still scattered among peripheral immunology, neuroscience and disease-specific models, and it is difficult to establish a unified transformation framework (151). Secondly, the distinction between central and peripheral effects is usually not clear, especially in the intervention of VNS or systemic cholinergic drugs (151). Third, the heterogeneity of disease and the difference of basic cholinergic function complicate the experimental design and result interpretation (152). Fourth, the influence of age and sex on cholinergic neuroimmune regulation is systematically understudied. Most preclinical experiments use young adult male rodents, and even when both sexes are included, data are rarely analyzed by sex. This gap limits the generalizability of findings and may contribute to the translational failure of cholinergic interventions (153). Finally, the relationship between receptor expression, microglial metabolic status and the outcome of functional inflammation has not been fully clarified, which limits the design ability of precision therapy (152).

Nevertheless, its transformation potential is still significant. In neurology, few anti-inflammatory paradigms can simultaneously act on neural circuits, glial cell biology and immune signals, and are conceptually compatible with precise intervention (154). Therefore, cholinergic regulation should not only be evaluated according to the maturity of the current evidence base, but also according to its ability to integrate emerging knowledge of microglia biology, immune metabolism and system neuroscience (155).

### 6.7 Toward a precision neuroimmune therapeutic framework

The long-term prospect of cholinergic neuroimmunotransformation lies in the shift from empirical stimulation or receptor targeting to precision neuroimmunomedicine (155). This framework should combine mechanism understanding, biological stratification and individualized intervention timing. It should recognize that different nervous system diseases may require different forms of cholinergic involvement: for example, neural regulation at the network level in stroke, inflammatory metabolic recalibration in neurodegeneration, or autonomic nervous immune stability in delirium and perioperative brain dysfunction (156). The framework should also include biomarkers to help judge not only whether inflammation exists, but also whether it can be regulated by cholinergic way.

The ultimate goal is not to simply inhibit inflammation, but to restore adaptive neuroimmune regulation. In this sense, cholinergic therapy may be best understood as a tool to reshape the set point of inflammation, rather than a means to directly inhibit immune function. The realization of this vision will depend on the resolution of a number of conceptual and methodological issues, which will be discussed in the next section.

## 7 Unresolved questions and future directions

Despite growing interest in cholinergic neuroimmune regulation, several conceptual and translational questions remain unresolved. A central issue is whether the classical cholinergic anti-inflammatory pathway remains sufficient as the main explanatory model for neuroinflammation in neurological disease (156). The original CAP framework was highly influential because it established that neural circuits can directly modulate inflammation. However, most neurological disorders involve a far more complex interplay of central cholinergic dysfunction, glial heterogeneity, barrier changes, peripheral immune signals, and age-related tissue remodeling than is captured by the traditional vagus-centered model (157). For this reason, the field may need to shift from viewing CAP as a single anti-inflammatory reflex to understanding cholinergic regulation as a multi-level neuroimmune network with disease-specific architecture. However, in order to analyze the respective roles of central and peripheral cholinergic pathways in disease-related contexts. This conceptual shift requires experimental validation through targeted interventions, which is a key priority for future research.

The second question that remains unanswered is the exact mechanism by which immune metabolism in microglia affects cholinergic activity (12). While numerous pieces of evidence show the relationship between cholinergic signaling, inflammatory signatures, oxidative stress, and mitochondrial adaptations, it is unclear whether metabolic adaptation is the driving force behind such effects or the secondary results of the process (12). It is crucial to differentiate the two factors since if metabolic adaptation is truly the determinant of immune reactivity, pharmacological targeting of receptors will be ineffective. Further studies are required to reveal whether cholinergic signaling influences microglial metabolic biases, including glycolytic metabolism, mitochondrial metabolism, lipid metabolism, and stress response adaptations, in aging, neurodegeneration, acute brain injury, and autoimmune conditions (48, 158). Future studies should use a combination of complementary approaches to address these questions. The first approach is to genetically perturb cholinergic receptors specifically in microglia. Researchers can do this, for instance, with a Cx3cr1-Cre-driven α7nAChR knockout. This method can establish causality. It allows scientists to separate cell-autonomous effects from systemic or neuronal contributions. The second approach involves longitudinal *in vivo* metabolic imaging. This method can track metabolic shifts in microglia in real time after cholinergic stimulation. Examples include two-photon NADH/FAD fluorescence lifetime imaging or 13C-glucose tracing coupled with magnetic resonance spectroscopy (MRS).The third approach combines single-cell metabolomics with spatial transcriptomics. Scientists can apply this to postmortem human tissue from patients with well-characterized cholinergic deficits. One example is Alzheimer’s disease patients with different degrees of basal forebrain degeneration. This work could reveal metabolic vulnerabilities that are specific to certain disease stages. Such vulnerabilities may be responsive to cholinergic modulation. The fourth approach uses pharmacological interventions in microglia-specific knockout models. These drugs could be selective AMPK activators, mTOR inhibitors, or HIF-1α stabilizers. Researchers can then test whether these metabolic sensors mediate the anti-inflammatory effects of α7nAChR signaling.

Another big issue is the heterogeneity of microglial cells states under different pathological conditions (48). The possibility of achieving identical effects of one and the same cholinergic manipulation on different biological processes appears to be increasingly small. Microglia in a geriatric brain, ischemic brain lesion, high levels of amyloids, and autoimmune plaque can significantly vary in their receptor composition, intracellular signaling pathways, metabolic state, and ability to heal tissues (159). Such heterogeneity raises a number of very important questions regarding the timing and specificity of the therapeutic action and appropriate patients’ stratification. The treatment, which has positive outcomes at the early stage of neuroinflammation, may turn out to be completely inefficient or even detrimental if it impairs the healing process (159). To target specific disease stages, we need to develop and validate reliable biomarkers. These biomarkers must be able to distinguish different microglial states in living patients. Several approaches are worth considering. First, we can use PET tracers. These tracers bind selectively to metabolic markers on microglia. For example, TSPO can label activated microglia. Newer tracers might target lipid-droplet-accumulating microglia (LDAM) or specific glycolytic enzymes. Second, we can use liquid biopsy assays. These assays measure microglia-enriched exosomal miRNAs or certain metabolites. Examples of such metabolites include itaconate and succinate. They reflect the metabolic programs happening inside the cells. Third, we should stratify patients in clinical trials by their baseline inflammatory-metabolic status. We can use markers such as CSF cytokine profiles or plasma lipid signatures. We should not rely on diagnosis alone. Biomarker-guided enrichment strategies of this kind could increase statistical power by a large margin. They could also uncover treatment effects that would otherwise stay hidden because of patient heterogeneity.

The distinction between the two types of mechanisms is yet to be made clear (160). Currently, many therapies that can be considered as cholinergic anti-inflammatory modulation, including vagus nerve stimulation and systemic cholinergic drugs, possess both central and peripheral activities. In cases involving neurological diseases, it is hard to assess whether the therapeutic effect is due to the regulation of CNS cells, modification of the tension of autonomic nerves, alleviation of systemic inflammation, or all three aspects combined (160). Understanding these multiple layers of action would not only allow us to understand the underlying mechanisms better, but is also crucial in the development of effective treatments, since the level of participation of central and peripheral systems might differ among various diseases (161). To separate central effects from peripheral ones, future studies need a set of complementary strategies. One strategy is selective peripheral vagotomy. Researchers can also block peripheral α7nAChR with quaternary antagonists. These drugs do not cross the blood–brain barrier. This approach isolates peripheral contributions. Another strategy is to deliver cholinergic agents directly into the central nervous system. This can be done by intracerebroventricular injection or convection-enhanced delivery. It bypasses peripheral effects and allows scientists to test central actions directly. Researchers can also compare CNS-penetrant cholinergic agonists with non-penetrant ones. They should use animal models that show disease-specific autonomic dysfunction. This comparison can reveal whether central or peripheral pathways matter more. Together, these approaches will clarify how therapeutic benefits arise in conditions like stroke or Alzheimer’s disease. Do these benefits come from direct modulation of brain microglia? Or do they come from secondary effects on systemic inflammation and autonomic tone?

Another limitation is the development of biomarkers (161). At present, there is no widely accepted biomarker framework that can reliably identify cholinergic neuroimmune dysfunction, track target participation and predict treatment response in nervous system diseases (162). Future progress is likely to depend on the integration of multi-layer information, including autonomic nerve indicators, humoral inflammatory markers, glial cell activation images, transcriptome characteristics and metabonomic spectrum (163). This multimodal biomarker pipeline is resource intensive. It has the potential to transform cholinergic neural regulation from a one size fits all approach to precise neuroimmunotherapy.

Emerging technologies provide important opportunities to address these gaps. Single cell transcriptomics and epigenomics can more accurately define disease-specific microglial status (163). Spatial transcriptomics can map the intersection of cholinergic signals and inflammatory niche in the diseased brain (76). Progress in metabonomics may clarify how cholinergic input reshapes cellular energy programs (76). At the same time, bioelectronic medicine and closed-loop nerve regulation methods may target the nerve immune loop more accurately than before (164). Together, these tools help to promote the field from descriptive relevance to mechanism based intervention. Importantly, these technologies can be directly applied to test the hypotheses outlined above: for example, spatial transcriptomics in animal models with microglia-specific cholinergic receptor deletion could reveal niche-specific metabolic dependencies; metabolomics in human cerebrospinal fluid stratified by APOE4 genotype could identify patient subgroups likely to benefit from α7nAChR-targeted therapies.

In the future, progress in this area will likely depend on three strategic shifts. First, cholinergic neuroimmune regulation should be studied as a systems problem rather than as a receptor-level pathway alone. Secondly, microglia status and immune metabolic background should be included in the main determinants of treatment response. Third, the clinical transformation should shift from empirical stimulation paradigm to biomarker guided, disease stage specific and age-/sex-stratified neuromodulation rather than empiric stimulation paradigms, recognizing that microglial metabolic states and cholinergic responsiveness differ substantially between young and aged brains and between males and females. If these goals can be achieved, cholinergic neuroimmune regulation may become a meaningful framework for precise intervention in neurology, rather than just a promising but not yet fully realized concept.

## 8 Conclusion

Cholinergic regulation of neuroinflammation has evolved from the concept of peripheral anti-inflammatory reflex to a broader framework for understanding neuroimmune communication in nervous system diseases. The evidence synthesized in this review supports a three-layered model in which microglia serve as the central cellular hub translating cholinergic signals into inflammatory outcomes, immunometabolism provides the mechanistic substrate that determines microglial responsiveness, and neuromodulation (pharmacological or bioelectronic) offers a translational avenue to target this axis. In this framework, the goal is not to suppress inflammation without discrimination. Instead, the goal is to restore adaptive neuroimmune regulation. This regulation must be calibrated to the stage of the disease. It must also be calibrated to the state of the cells, and to a person’s genetic and metabolic background.

Several specific priorities should guide future research. First, we need to test directly whether cholinergic signaling modulates the AMPK, mTOR, and HIF-1α pathways in microglia. We also need to test whether it shifts the balance between glycolysis and mitochondrial respiration. Such testing requires microglia-specific genetic perturbations. It also requires longitudinal metabolic imaging in animal models. Second, we must develop strategies for patient stratification. These strategies should include biomarkers of the microglial metabolic state. Examples include TSPO PET, CSF metabolites, and exosomal miRNAs. They should also include biomarkers of cholinergic tone, such as heart rate variability or acetylcholinesterase activity. With these tools, researchers can design clinical trials that are specific to disease stage and subtype. Third, the field must systematically address gaps in our knowledge about aging and sex. Both factors substantially alter how microglia respond. Yet they are rarely accounted for in current experimental designs. Fourth, non-α7 nicotinic receptors and muscarinic receptors deserve dedicated mechanistic and translational studies. The contributions of astrocytes deserve the same level of attention. By bringing together cholinergic biology, microglial heterogeneity, immunometabolism, and systems neuroscience, the field can move forward. It can move toward precision neuroimmune interventions. These interventions would recalibrate inflammatory set points in a way that is sensitive to the surrounding context.
